# Genetic Diversity Analysis of Surface-Related Antigen (SRA) in *Plasmodium falciparum* Imported From Africa to China

**DOI:** 10.3389/fgene.2021.688606

**Published:** 2021-08-05

**Authors:** Bo Yang, Hong Liu, Qin-Wen Xu, Yi-Fan Sun, Sui Xu, Hao Zhang, Jian-Xia Tang, Guo-Ding Zhu, Yao-Bao Liu, Jun Cao, Yang Cheng

**Affiliations:** ^1^Laboratory of Pathogen Infection and Immunity, Department of Public Health and Preventive Medicine, Wuxi School of Medicine, Jiangnan University, Wuxi, China; ^2^Center for Global Health, School of Public Health, Nanjing Medical University, Nanjing, China; ^3^Key Laboratory of National Health and Family Planning Commission on Parasitic Disease Control and Prevention, Jiangsu Provincial Key Laboratory on Parasite and Vector Control Technology, Jiangsu Institute of Parasite Diseases, Wuxi, China

**Keywords:** *Plasmodium falciparum*, PfSRA, genetic diversity, imported malaria cases, DNA sequencing, Jiangsu Province

## Abstract

*Plasmodium falciparum* surface-related antigen (SRA) is located on the surfaces of gametocyte and merozoite and has the structural and functional characteristics of potential targets for multistage vaccine development. However, little information is available regarding the genetic polymorphism of *pfsra*. To determine the extent of genetic variation about *P. falciparum* by characterizing the *sra* sequence, 74 *P. falciparum* samples were collected from migrant workers who returned to China from 12 countries of Africa between 2015 and 2019. The full length of the *sra* gene was amplified and sequenced. The average pairwise nucleotide diversities (π) of *P. falciparum sra* gene was 0.00132, and the haplotype diversity (*Hd*) was 0.770. The average number of nucleotide differences (*k*) for *pfsra* was 3.049. The ratio of non-synonymous (*dN*) to synonymous (*dS*) substitutions across sites (*dN*/*dS*) was 1.365. Amino acid substitutions of *P. falciparum* SRA could be categorized into 35 unique amino acid variants. Neutrality tests showed that the polymorphism of PfSRA was maintained by positive diversifying selection, which indicated its role as a potential target of protective immune responses and a vaccine candidate. Overall, the ability of the N-terminal of PfSRA antibodies to evoke inhibition of merozoite invasion of erythrocytes and conserved amino acid at low genetic diversity suggest that the N-terminal of PfSRA could be evaluated as a vaccine candidate against *P. falciparum* infection.

## Introduction

Malaria has been a major global health concern of humans throughout history and is a leading cause of disease and death across many tropical and subtropical countries. In 2019, an estimated 229 million malaria cases and 409,000 malaria-caused deaths globally were reported (WHO, 2020). Among the five species of *Plasmodium* that infect humans, *P. falciparum* infection causes the highest mortality and morbidity and the most serious clinical symptoms (Buffet et al., 2011).

The resurgence and spread of antimalarial drug resistance (Ménard et al., 2015; WWARN, 2015) along with vector resistance to insecticides (Dhiman and Veer, 2014; Strode et al., 2014) have the potential to reduce the impact of existing malaria control strategies and make vaccines a public health priority. Although extensive studies have been conducted on several blood-stage antigens, few have shown the quality required for a candidate vaccine. In a systematic screen of uncharacterized *P. falciparum* proteins for potential blood-stage vaccine candidates, using data from transcriptome studies of *P. falciparum*, data-mining analysis of the genes with peak mRNA expression levels in late schizogony was performed (Bozdech et al., 2003; Le Roch et al., 2003) and another study on the prediction of PfSUB-1 protease specificity (Gilson et al., 2006). The results showed that *P. falciparum* surface-related antigen (PfSRA) emerged as the top hit with both signal peptide and a predicted glycosylphosphatidylinositol (GPI) attachment site. PfSRA is localized on the surfaces of both gametocytes and merozoites. The processed 32-kDa PfSRA protein fragment binds normal human erythrocytes. Immunoepidemiological studies in malaria-infected populations suggest the presence of naturally acquired protective antibodies against PfSRA. Parasite growth inhibition assays indicated that the antibodies against PfSRA could potently inhibit the invasion of merozoite on erythrocytes. Overall, the structural and functional characteristics of PfSRA indicate that it would be a promising vaccine target (Amlabu et al., 2018).

The low protective efficacy of vaccines against clinical malaria has been in part limited by extensive genetic diversity, which enables parasites to evade human immune responses and may lead to vaccine failure (Takala et al., 2002). However, the evidence for *pfsra* genetic diversity is limited. Therefore, it is necessary to study the characteristics of *pfsra* toward finding suitable vaccine candidates and understanding its population genetic structure. Accordingly, this study analyzed the full-length sequence of *sra* from the *P. falciparum* collected from infected migrant workers returning to the Jiangsu Province from Africa. We determined the nucleotide divergence and polymorphisms level of *sra* sequences to trace signatures of selection and to determine the extent of genetic variation in *P. falciparum* by characterizing the *sra* sequence at the nucleotide and protein levels.

## Materials and Methods

### Study Areas and Blood Samples Collection

The samples of *P. falciparum* were obtained from febrile patients in Jiangsu Province, China, from 2015 to 2019, who had returned from working in tropical regions of sub-Saharan Africa endemic for malaria (Chu et al., 2018). A total of 74 *P. falciparum*-infected blood samples were collected from 12 countries. The subjects were identified for mono-infection of *P. falciparum* by microscopic examination of blood smears stained with Giemsa. The isolates were identified by specific polymerase chain reaction (PCR).

### Amplification and Sequencing Analysis of *pfsra*

The full-length nucleotide sequences of *sra* from *P. falciparum* were divided into four fragments and amplified by PCR with primers designed as *pfsra*-1-Forward (5′-ATG TTT CTA AGT TCT AAG AAA AGA A-3′) and *pfsra*-1-Reverse (5′-AAA GGA ATC TGT CTC ATT ATT TGT T-3′), *pfsra*-2-Forward (5′-GAT AAT GAA GAA ACA GAA GAT ATT G-3′), and *pfsra*-2-Reverse (5′-ATC TAA TAG TTG TAT ATA AGC ATA TTT ATT AAC-3′), *pfsra*-3-Forward (5′-AAT AAG AAT TCA AAT CAA TCA TAT AAT T-3′) and *pfsra*-3-Reverse (5′-ATA ATA TTT CCT CAC AAT TTT TAC ATG-3′), and *pfsra*-4-Forward (5′-GTA CCT GCC AAA ATT AAA TAT ATA GAA-3′) and *pfsra*-4-Reverse (5′-TTA ATA TAT CGA AAT AAA TAT CAT AAG-3′), respectively. The *pfsra* (PlasmoDB, PF3D7_1431400) sequence from the *Plasmodium* Genomics Resource database was used as the reference gene sequence. The PCR amplification reactions were performed in a volume of 50 μl including 100 ng of genomic DNA, 0.2 μM each of the forward and reverse primers, 0.2 mM deoxynucleoside triphosphate, 2.5 units of DNA polymerase in 1 × *FastPfu* buffer (*TransStart*^®^ *FastPfu* DNA polymerase, Beijing, China), and nuclease-free water up to 50 μl. The PCR amplification of *pfsra* genes was carried out in Mastercycler (Eppendorf, Hamburg, Germany). Amplification was performed as follows: denaturation at 95°C for 2 min, 35 cycles of 95°C for 20 s, 50°C for 20 s, and 65°C for 1 min, and final extension at 65°C for 5 min. The PCR products were analyzed using 1% agarose gel electrophoresis, stained with SuperStain (CWBIO, Jiangsu, China), and visualized by ultraviolet transilluminator (Bio-Rad ChemiDoc MP, Hercules, United States). The lengths of the PCR products were estimated based on their mobility relative to a standard DNA marker (TransGen Biotech, Beijing, China). Sequencing reactions were performed using GENEWIZ (Suzhou, China) with an ABI 3730xl DNA Analyzer (Thermo Fisher Scientific, Waltham, United States). All 74 samples generated a single amplification fragment of the expected size, and direct Sanger DNA sequencing of the forward and reverse directions was conducted to ensure the accuracy of the obtained sequences.

### Sequence Alignment and Genetic Data Analysis

The geographical distribution map of *P. falciparum* samples was constructed by Arcgis10.2 software (ESRI, 2011). In order to evaluate diversity, *pfsra* sequence was used as template and aligned using GeneDoc2.7.0.^1^ The primary structure of the PfSRA protein was demonstrated by UniProt.^2^ The nucleotide sequences of *pfsra* were translated into the deduced amino acid (aa) sequences by DNASTAR (Burland, 2000). The predicted amino acid sequences of PfSRA from the PCR sequenced genomic fragments were aligned with the sequence of *P. falciparum* genome strain 3D7 by the MUSCLE algorithms in the MEGA 7.0 program (Kumar et al., 2016). A logo plot for each *pfsra* population was constructed to analyze the polymorphic characteristics of PfSRA by the WebLogo program.^3^ In addition, a codon-based test of purifying selection was analyzed by MEGA 7.0 program (Kumar et al., 2016). The non-synonymous mutations (*dN*), synonymous mutations (*dS*), and the *dN*/*dS* ratio from MEGA 7.0 were tested and compared by the *Z*-test (*p* < 0.05) using Nei and Gojobori’s method, corrected by Jukes and Cantor and 1,000 bootstrap replications (Nei and Gojobori, 1986). Under purifying selection, *dN* will be less than *dS* (*dN*/*dS* < 1), while when the positive selection is more advantageous, *dN* will exceed *dS* (*dN*/*dS* > 1).

The average pairwise nucleotide diversity (π), number of haplotypes (*H*), and haplotype diversity (*Hd*) were calculated by DnaSP v6 (Rozas et al., 2017). The nucleotide diversity was analyzed by DnaSP v6 with a window length of 100 base pairs (bp) and a step size of 25 bp. In addition, the neutrality tests (Tajima’s *D*, Fu and Li’s *D*^∗^, and Fu and Li’s *F*^∗^) implemented in DnaSP v6 software were utilized to measure the departure of the neutral mode prediction of molecular evolution (Fu and Li, 1993; Tajima, 1993). In order to determine the evolutionary relationship of the aligned sequences, based on nucleotide sequences, the phylogenetic tree of *sra* was constructed with the neighbor-joining method in MEGA 7.0. The *sra* sequences of diverse malaria parasites species included the SRA haplotypes of *plasmodium* from humans, non-human primate, avian, and murine malaria, which were obtained from the PlasmoDB and NCBI databases.

## Results

### Geographical Origin of *P. falciparum*

**FIGURE 1:**
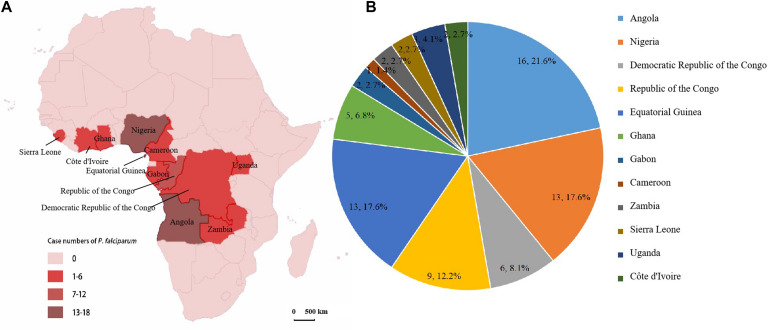
Origins of malaria cases were imported from 12 countries of sub-Saharan Africa to the Jiangsu Province of China. **(A)** A map of Africa showing the countries of origin of *P. falciparum* isolates. **(B)** The total number of genotyped samples per region and percentage of samples.

A total of 74 clinical isolates of *P. falciparum* showed the geographical distribution in 12 sub-Saharan Africa countries. These isolates were mainly from the west coast of Africa, including Angola (*n* = 16, 21.6%), Nigeria and Equatorial Guinea (*n* = 13, 17.6%), and the Republic of the Congo (*n* = 9, 12.2%) (Figure 1). Of the 74 sequencing samples, 3 were from Eastern Africa (Uganda), 22 were from Western Africa (Sierra Leone, Côte d’Ivoire, Ghana, and Nigeria), 31 were from Central Africa (Cameroon, Equatorial Guinea, Gabon, Republic of the Congo, and Democratic Republic of the Congo), and 18 were from Southern Africa (Zambia and Angola). Overall, 74 cases of *P. falciparum* infection were identified in our study (Table 1). Full details for the isolates were provided (Supplementary Table 1).

**TABLE 1:** Origin of imported *Plasmodium falciparum* in 2015–2019.

Country	2015 number	2016 number	2017 number	2018 number	2019 number	Total number (%)
Angola	3	3	2	4	4	16 (21.6%)
Nigeria	2	3	4	3	1	13 (17.6%)
Democratic Republic of the Congo	3	0	0	2	1	6 (8.1%)
Republic of the Congo	3	2	0	1	3	9 (12.2%)
Equatorial Guinea	7	1	2	2	1	13 (17.6%)
Ghana	2	0	1	2	0	5 (6.8%)
Gabon	1	0	0	1	0	2 (2.7%)
Cameroon	0	1	0	0	0	1 (1.4%)
Zambia	0	0	0	1	1	2 (2.7%)
Sierra Leone	0	1	0	0	1	2 (2.7%)
Uganda	0	1	0	0	2	3 (4.1%)
Côte d’Ivoire	0	0	0	0	2	2 (2.7%)
Total	21	12	9	16	16	74 (100%)

### Characterization of PfSRA

**FIGURE 2:**
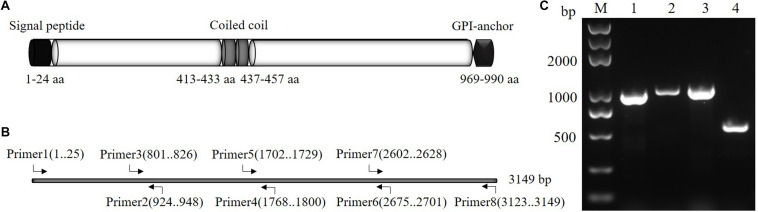
Predicted *P. falciparum* SRA protein primary structure and primer design diagram. **(A)** Diagram of PfSRA protein primary structure. **(B)** Diagram of *pfsra* primers design. **(C)** Amplification of four fragments of *pfsra* by PCR. Abbreviations: M, DNA marker; 1, the first fragment of *pfsra* (948 bp); 2, the second fragment of *pfsra* (1 kb); 3, the third fragment of *pfsra* (1 kb); 4, the fourth fragment of *pfsra* (548 bp).

The length of SRA encoded by the *pfsra* full-length was 990 (aa), beginning with the predicted 24-aa signal peptide sequence (aa 1–24) and ending with a GPI-anchor (aa 969–990). Other specific regions, such as two coiled-coil regions, were also identified in the predicted protein primary structure of *P. falciparum* (aa 413–433 and 437–457) (Figure 2A). The full-length nucleotide sequences of *sra* from *P. falciparum* was 3,149 bp and was amplified by PCR using primer 1 (1–25 bp), primer 2 (924–948 bp), primer 3 (801–826 bp), primer 4 (1,768–1,800 bp), primer 5 (1,702–1,729 bp), primer 6 (2,675–2,701 bp), primer 7 (2,602–2,628 bp), and primer 8 (3,123–3,149 bp) (Figure 2B).

### Nucleotide Polymorphism of *pfsra*

**FIGURE 3:**
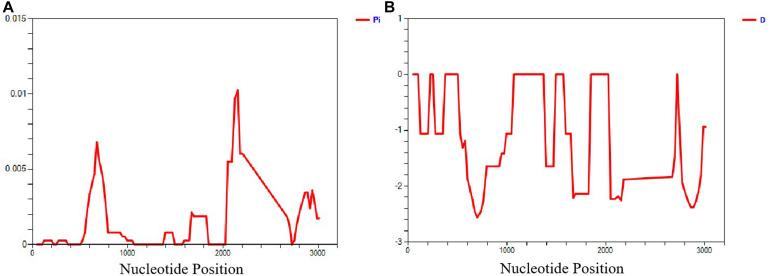
Sliding window plot analyses show the sequence diversity (π) and Tajima’s *D.* **(A)** Sequence diversity of PfSRA. **(B)** Tajima’s *D* of PfSRA.

**FIGURE 4:**
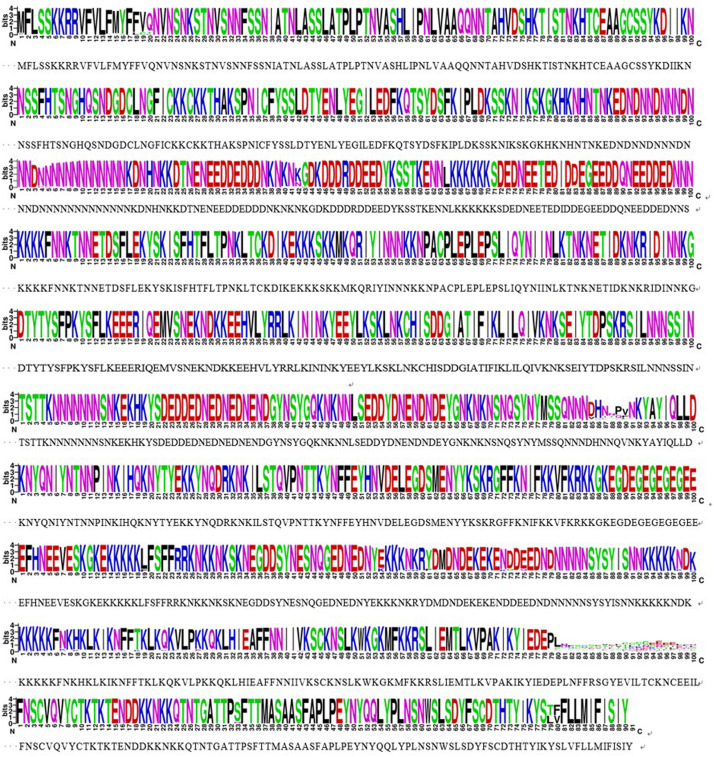
Conservative locus analysis of the PfSRA amino acid sequences defined by WEBLOGO. Each logo consists of stacks of symbols, and each position in the sequence corresponds to stacks of symbols. The height within the stack of each individual amino acid abbreviation indicates its relative frequency at that specific position.

The *sra* genes of 74 *P. falciparum* isolates were successfully amplified by PCR, corresponding to nucleotides 1–948, 801–1,800, 1,702–2,701, and 2,602–3,149, respectively, and a single PCR product with an expected size of 948 bp (*pfsra1*), 1 kb (*pfsra2* and *pfsra3*), and 548 bp (*pfsra4*) (Figure 2C). The direct sequencing of the purified PCR fragments indicated that there were no superimposed signals on the electropherograms of *pfsra.* Compared with the reference 3D7 strain, 74 isolates (100%) showed non-synonymous mutation. Overall, 63 single nucleotide polymorphisms (SNPs) were found in 74 isolates with the average π value of 0.00132 for *pfsra*. The sliding method plot using DnaSP v6 with a window length of 100 bp and a step size of 25 bp showed that the π value of *pfsra* is in the range of 0–0.01023. The conservative regions of 0–0.6 and 0.8–1.6 kb were observed in *pfsra* with π values of 0 approximately (Figure 3). The average number of nucleotide differences (*k*) of *pfsra* was 3.049. Nucleotide diversity of *pfsra* was categorized into 35 distinct haplotypes, and the estimated *Hd* was 0.770 (Table 2). For amino acid, the frequencies and types of mutation in the full-length of PfSRA (aa 1–990) were briefly presented in Figure 4. The C-terminal fragments of the PfSRA (aa 585–591, aa 879–900) showed relatively high polymorphism. More detailed amino acid comparison results are reported in Supplementary Figure 1.

**TABLE 2:** Estimates of number of haplotypes, haplotype diversity, nucleotide diversity, and neutrality indices of *pfsra.*

Region	No. of samples	No. of haplotypes	*Hd*	*dN*/*dS*	Diversity ± SD		Tajima’s *D*	FU and Li’s D*	FU and Li’s F*
					Nucleotide	Haplotype			
Eastern Africa	3	3	1	1.579	0.00437 ± 0.00092	1 ± 0.177	–0.73807	–0.73807	–0.77178
Western Africa	21	21	0.996	1.313	0.00252 ± 0.00072	0.996 ± 0.014	–2.10441	–3.02686	–3.21120
Central Africa	30	25	0.968	1.267	0.00208 ± 0.00073	0.968 ± 0.024	–2.34218	–3.94714	–4.03442
Southern Africa	18	15	0.977	1.252	0.00186 ± 0.00086	0.977 ± 0.027	–2.42786	–3.45933	–3.66946
Total	74	35	0.770	1.365	0.00132 ± 0.00078	0.770 ± 0.054	–2.75387	–6.85882	–6.25597

In DnaSP software, D* and F* tests are based on the neutral model prediction. This command calculates the statistical tests D* and F* proposed by Fu and Li (1993) for testing the hypothesis that all mutations are selectively neutral (Kimura, 1983).

### Genetic Population Structure of *pfsra*

Based on the average values of *dS* and *dN*, the population genetic structure of the *P. falciparum* samples was analyzed using the *sra* gene polymorphisms in the codon-based purifying selection test. Results showed that there was diversifying selection or positive selection in *P. falciparum sra* population (*dS* − *dN* = −0.00075). In addition, the mean ratio of across sites non-synonymous (*dN*) to synonymous (*dS*) substitutions (*dN*/*dS*) was 1.365, and most of the nucleotide substitutions detected were non-synonymous, which also showed that the genetic variations of *pfsra* were maintained by positive selection. Tajima’s *D* and Fu and Li’s *D*^∗^ and *F*^∗^ tests rejected a neutral polymorphism occurrence model with values of *pfsra* (Tajima’s *D* = −2.75387, *p* < 0.05, Fu and Li’s *D*^∗^ = −6.85882, *p* < 0.05, and Fu and Li’s *F*^∗^ = −6.25597, *p* < 0.05) (Table 2). Full details for all study countries were provided (Supplementary Table 2).

### Phylogenetic Analysis of *sra*

**FIGURE 5:**
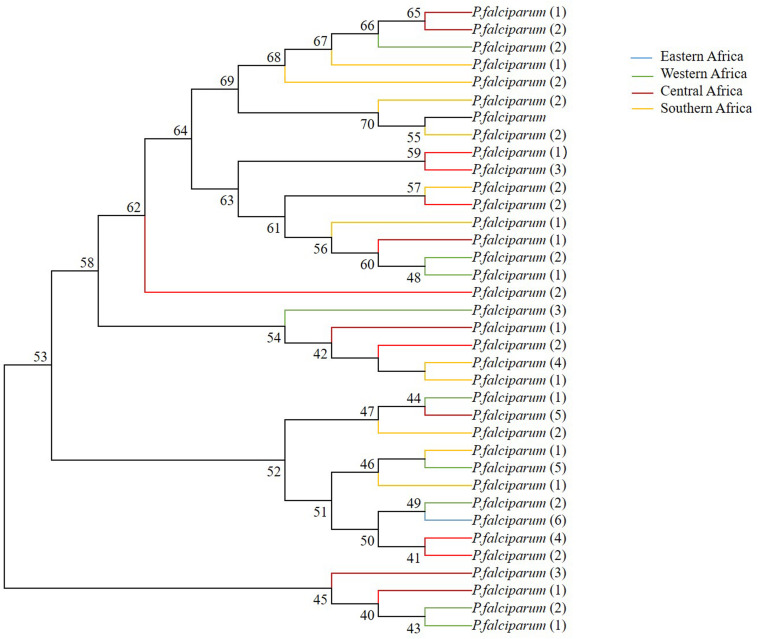
Phylogenetic relationship of *sra* full-length genes within *pfsra* sequences based on the neighbor-joining method. Numbers at nodes show bootstrap values.

As predicted based on the signature of positive selection and the level of genetic diversity described above, the phylogenetic relationship among 35 distinct haplotypes was detected in the *pfsra* sequences (1 was from Eastern Africa; 9 were from Western Africa; 14 were from Central Africa; and 11 were from Southern Africa) (Figure 5). The phylogenetic tree of 11 alleles of *sra* gene of 11 species of human and non-human *Plasmodium* primates was constructed by the neighbor-joining method (Supplementary Figure 2). Supplementary Table 3 provides the *sra* gene ID number and gene length of other malaria parasite species.

## Discussion

Apart from the complex life cycle of the malaria parasite involving the mosquito vector and human host, the malaria parasite exhibits extensive antigenicity and genetically diverse stages that may pose an adverse obstacle to malarial control strategies. Thus, a deeper understanding of patterns and mechanisms of sequence variation and genetic recombination may contribute to the design of a vaccine that represents the global repertoire of polymorphic malaria surface antigens (Bharti et al., 2012). Systematic screens for uncharacterized *P. falciparum* invasion-related proteins evaluated PfSRA as one of the top hits that emerged; it contains coiled-coil domains known to be less polymorphic (Villard et al., 2007; Kulangara et al., 2009; Amlabu et al., 2018). Our investigation into the extent of sequence variation is consistent with this. In addition, coiled-coil domains form a stable structure, which elicit functional antibodies, thus blocking the related domains in many organisms and were considered to be the basis for the chemical synthesis of three PfSRA peptides designed to generate antibodies (Tripet et al., 2006; Gustchina et al., 2013; Jiang et al., 2016; Amlabu et al., 2018). Furthermore, these domains have been evaluated as potential targets for immunotherapy such as peptide-based vaccine strategies (Stoute et al., 1995; Demangel et al., 1998; Adda et al., 1999). We analyzed the full-length of *pfsra* (74 isolates) and found that the C-terminal fragments of the *pfsra* (π = 0.00198) show polymorphism probably due to selection pressure. Comparatively, the N-terminal of *sra* is a relatively conserved sequence (π = 0.00083).

A previous study suggested that people infected with malaria have naturally acquired antibodies against PfSRA and PfSRA N-terminal antibodies could partially inhibit merozoite invasion of erythrocytes by parasite growth inhibition assays (Amlabu et al., 2018). Now, the evidence of relatively conservative N-terminus might raise the possibility that it has the potential to be a candidate for anti-malarial vaccine. The ratio of non-synonymous (*dN*) to synonymous (*dS*) substitutions across sites was used as an index to evaluate selection pressure; *dN*/*dS* > 1 indicates diversifying positive selection. Further neutrality tests were carried out to determine the types and characteristics of natural selection on the *pfsra*. Statistically significant negative values of neutrality tests suggest an excess of rare polymorphisms in the population and provide evidence of purifying or directional (positive) selection (Fu and Li, 1993; Akey et al., 2004). The phylogenetic tree of 11 alleles of *sra* gene of 11 species showed that *pfsra* and other species occupied distinct bifurcating branches, supporting an ancient divergence times of the malarial parasite lineage.

The nucleotide diversity of *pfsra* in Southern Africa (π = 0.00186 ± SD 0.00086), Central Africa (π = 0.00208 ± SD 0.00073), and Western Africa (π = 0.00252 ± SD 0.00072) was lower than that in Eastern Africa (π = 0.00437 ± SD 0.00092), which may be related to the higher transmission rate of *P. falciparum* in Eastern Africa. Furthermore, more samples are needed in future research to support our findings and to control the limitations of small sample size (large confidence interval) in a single area. A previous study had also shown that *P. falciparum* has a spectrum of population structure: linkage “equilibrium,” low levels of differentiation and high diversity in regions with high levels of transmission (Anderson et al., 2000; Huang et al., 2020). Mutation, recombination, gene flow, and natural selection may contribute to the genetic diversity of malaria parasites (Cole-Tobian and King, 2003).

In the analysis of *pfsra* full-length, there were abundant polymorphisms found. Samples from the four Africa regions showed their own distinct diversity patterns. Interestingly, two larger-size parasite population (Western Africa and Central Africa) showed more polymorphisms compared to those in Eastern Africa and Southern Africa. Some mutations showed the regional differences based on the geographical isolation effect; for example, the 15th amino acid mutant (M15Y) only occurred in Central Africa; K333E was only found in Western Africa. These phenomena indicate that it is necessary to continuously monitor these regional characteristic mutations in order to explore their association with regional malaria epidemics. Overall, apart from the conserved N-terminus, the composition of PfSRA vaccine should consider the high-frequency alleles instead of the C-terminus of wild-type ones (Conway, 2007).

Epidemiological studies have indicated that the level of heterologous mating in malaria populations is positively correlated with the prevalence of mixed allele infections and transmission rates (Chenet et al., 2008). The generation of relevant genetic, immunologic, and epidemiologic data for the *sra* gene is necessary, especially in areas with low malaria endemicity. Even in geographical areas with low transmission, the development of vaccine strategies should include results of diversity analysis. The uneven geographical distribution of alleles may jeopardize the development and use of vaccines targeting specific variable site, as local variation may not be taken into account in vaccine design (Cole-Tobian and King, 2003). The study of different genes and their alleles is helpful for us to understand the trends of genetic variation and if alleles could render vaccine ineffective. Given the genetic diversity found in the region, an alternative to improve the vaccine effectiveness is to create a construct with the most common region-specific alleles (Cole-Tobian and King, 2003; Chenet et al., 2008).

## Conclusion

The C-terminal fragments of the *sra* gene of *P. falciparum* showed polymorphism due to positive diversifying selection, which would hinder SRA-based vaccine development. Comparatively, in addition to the coiled-coil domains that have been evaluated as potential targets of peptide-based vaccines previously, the conserved N-terminal of *pfsra* is also a promising vaccine candidate against *P. falciparum* infection.

## Data Availability Statement

The datasets presented in this study can be found in online repositories. The names of the repository/repositories and accession number(s) can be found in the article/Supplementary Material.

## Ethics Statement

This study was approved by the Ethics Committee of Jiangsu Institute of Parasitic Diseases (JIPD) (IRB00004221), Wuxi, China. The patients/participants provided their written informed consent to participate in this study.

## Author Contributions

BY and YC conceptualized the study, wrote the manuscript and contributed to the interpretation of the data. BY, HL, Q-WX, SX, HZ, J-XT, and G-DZ collected and analyzed the samples. Y-FS performed statistical and bioinformatics analysis. BY, YC, Y-BL, and JC revised the manuscript critically for important intellectual content. All the authors contributed to this article and approved the submitted version.

## Conflict of Interest

The authors declare that the research was conducted in the absence of any commercial or financial relationships that could be construed as a potential conflict of interest.

## Publisher’s Note

All claims expressed in this article are solely those of the authors and do not necessarily represent those of their affiliated organizations, or those of the publisher, the editors and the reviewers. Any product that may be evaluated in this article, or claim that may be made by its manufacturer, is not guaranteed or endorsed by the publisher.

## Footnotes

1
https://github.com/karlnicholas/GeneDoc

2
https://www.uniprot.org/uniprot/Q8ILF1

3
https://weblogo.berkeley.edu/logo.cgi

## Supplementary Material

Supplementary Figure 1 Amino acid sequence alignment of *P. falciparum* SRA. Gray area indicates that amino acid is identical across all aligned sequences.

Supplementary Figure 2 Neighbor-joining tree of 11 unique alleles encoded by *sra* from 11 *Plasmodium* parasite species. Numbers at nodes show bootstrap values.

Supplementary Table 1 Country of origin and parasitemia of *P. falciparum* samples.

Supplementary Table 2 Neutrality tests of *pfsra* among 74 *P. falciparum* isolates.

Supplementary Table 3 The *sra* Gene ID number and gene length of other *Plasmodium* species.
