# Supplementary material for: Genetic Diversity Analysis of Surface-Related Antigen (SRA) in Plasmodium falciparum Imported From Africa to China
Source: Front Genet. 2021 Aug 5;12:688606. doi: 10.3389/fgene.2021.688606 (PMC8378275; doi:10.3389/fgene.2021.688606)
Supplement: Supplementary Figure 1 — Amino acid sequence alignment of P. falciparum SRA. Gray area indicates that amino acid is identical across all aligned sequences. [file Image_1.pdf]

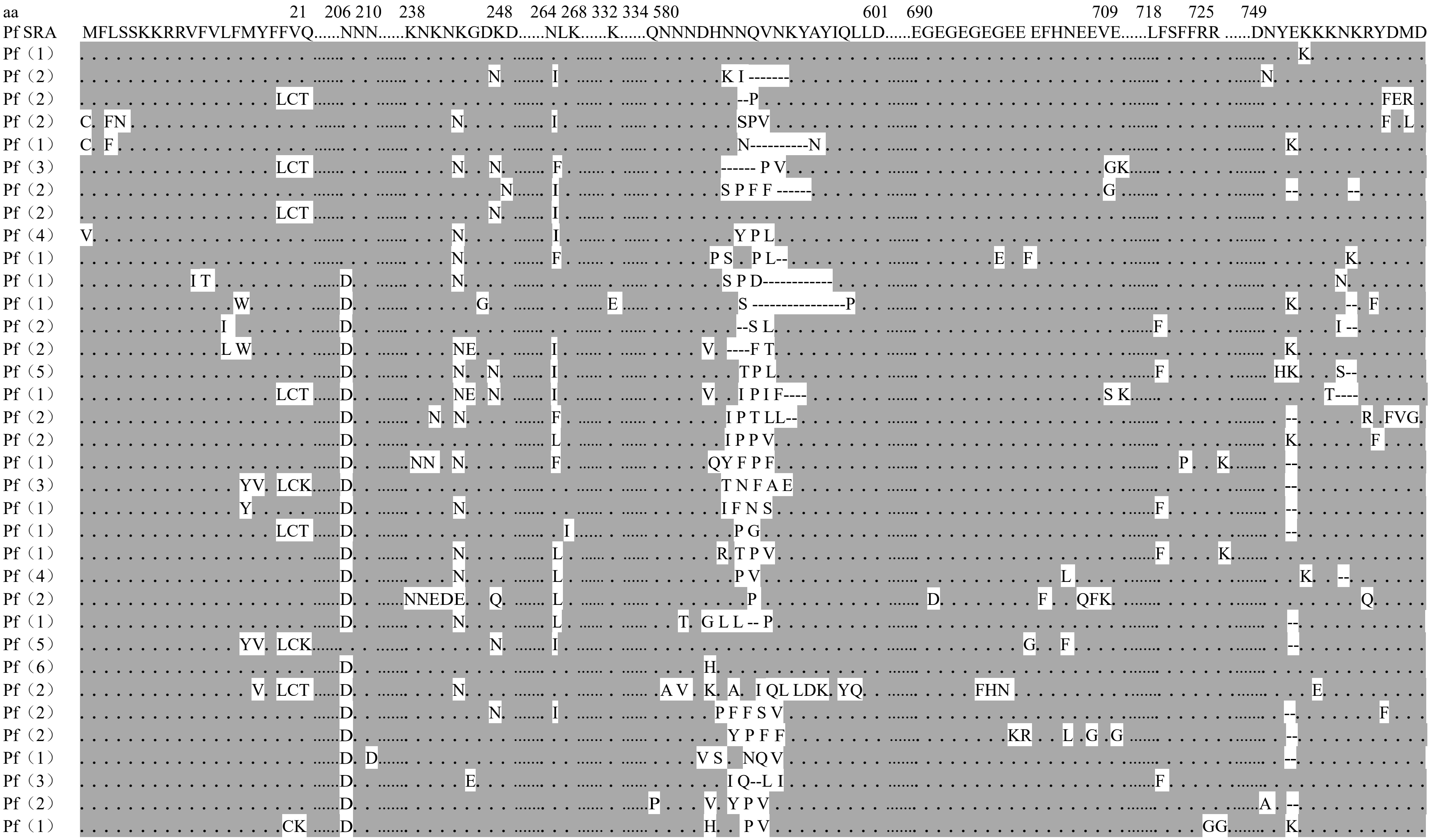
aa
21 206 210 238 248 264 268 332 334 580 601 690 709 718 725 749
Pf SRA MFLSSKKRRVFVLFMYFFVQ......NNN......KNKNKGDKD......NLK......K......QNNNDHNNQVNKYAYIQLLD......EGEGEGEGEE EFHNEEVE......LFSFFRR ......DNYEKKKNKRYDMD

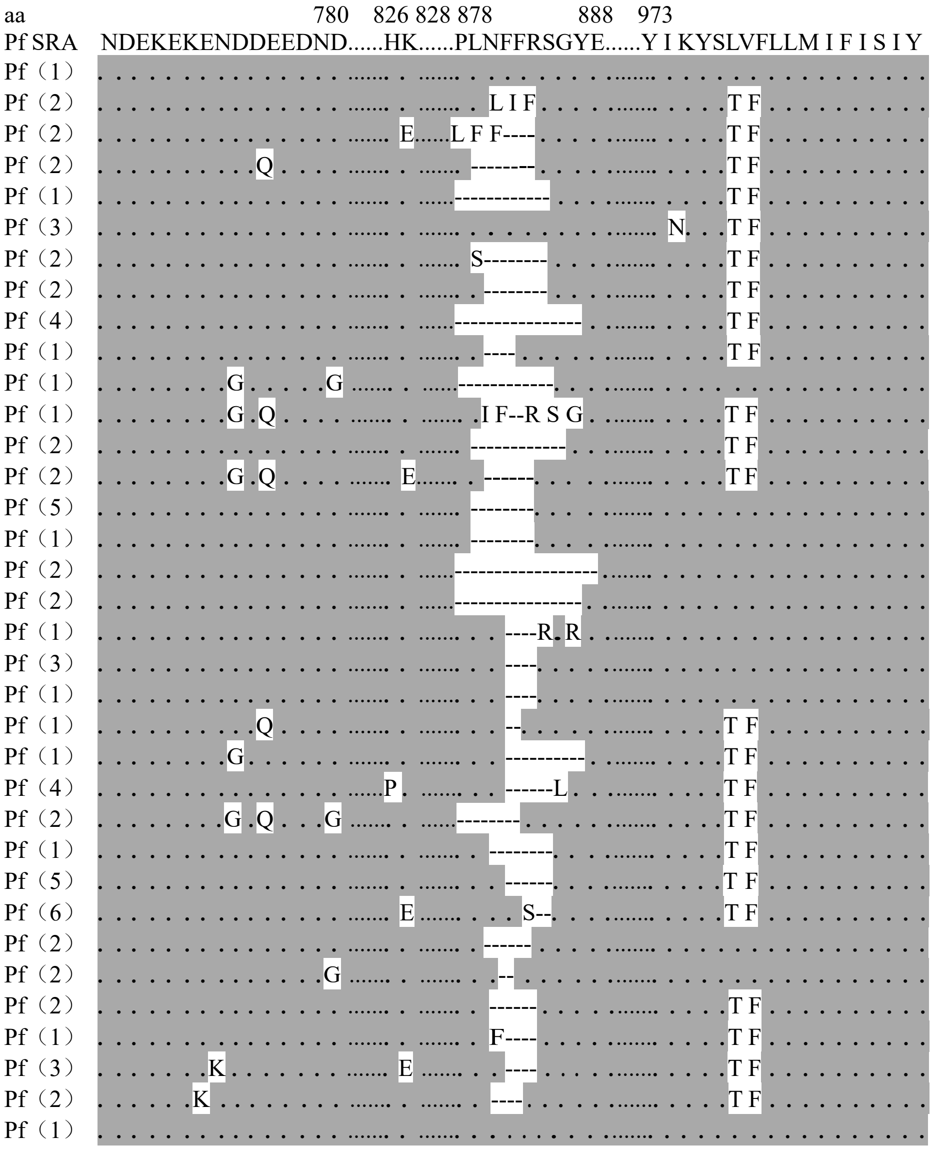

Supplementary Figure 1. Amino acid sequence alignment of *P.falciparum* SRA. Gray area indicates that amino acid is identical across all aligned sequences.
