# Supplementary figures and images for: Genetic Diversity Analysis of Surface-Related Antigen (SRA) in Plasmodium falciparum Imported From Africa to China
Source: Front Genet. 2021 Aug 5;12:688606. doi: 10.3389/fgene.2021.688606 (PMC8378275; doi:10.3389/fgene.2021.688606)

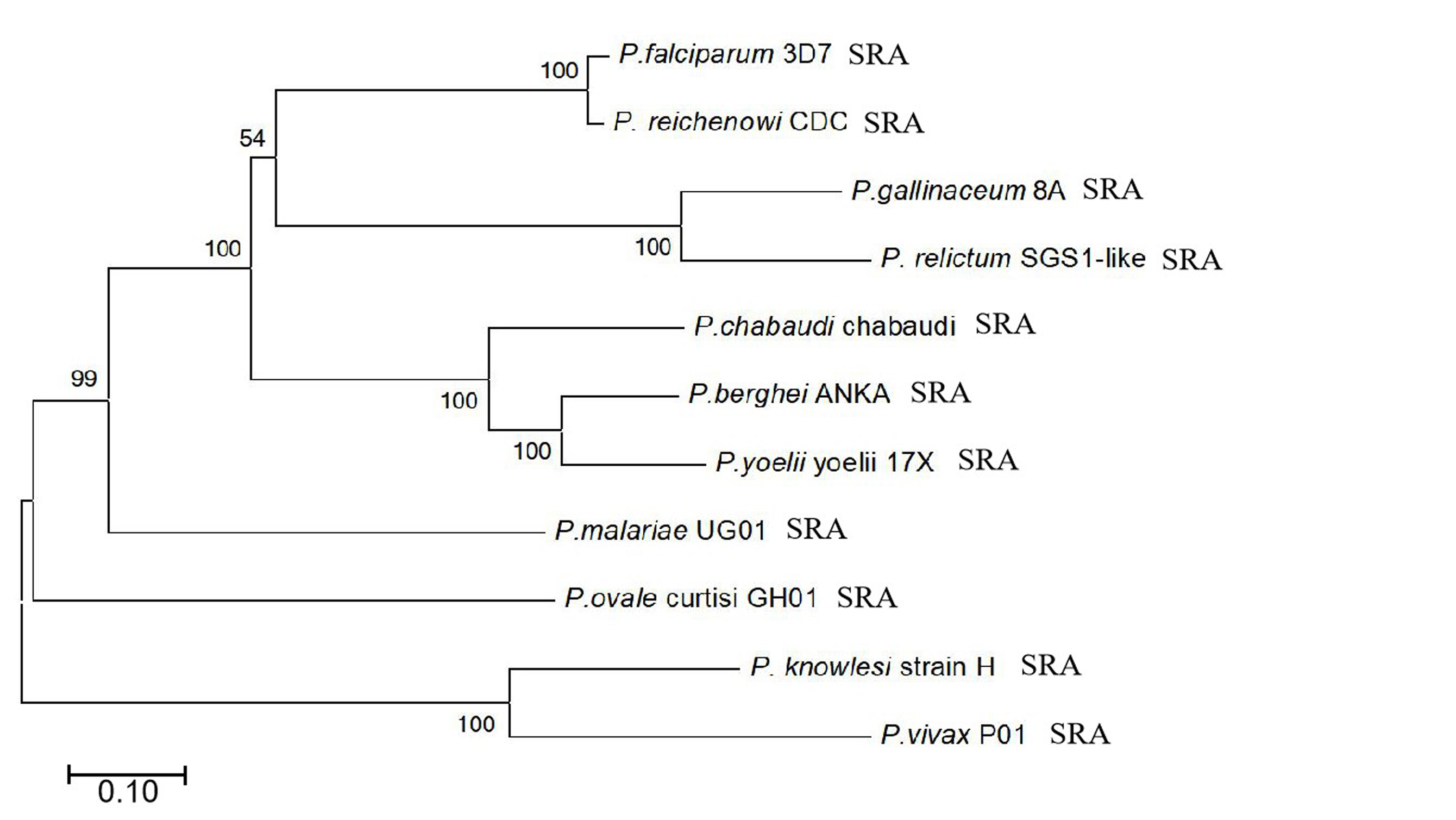

Supplement: Supplementary Figure 2 — Neighbor-joining tree of 11 unique alleles encoded by sra from 11 Plasmodium parasite species. Numbers at nodes show bootstrap values. [file Image_2.tif]
