# Supplementary material for: Genetic Diversity Analysis of Surface-Related Antigen (SRA) in Plasmodium falciparum Imported From Africa to China
Source: Front Genet. 2021 Aug 5;12:688606. doi: 10.3389/fgene.2021.688606 (PMC8378275; doi:10.3389/fgene.2021.688606)
Supplement: Supplementary Table 1 — Country of origin and parasitemia of P. falciparum samples. [file Table_1.docx]

**Table S1** Country of origin and parasitemia of *P. falciparum* samples

| Isolate number | Country of origin | City | Parasitaemia | GenBank accession number |
| --- | --- | --- | --- | --- |
| Pf(A1)  Pf(A2)  Pf(A3)  Pf(A4)  Pf(A5)  Pf(A6)  Pf(A7)  Pf(A8)  Pf(A9)  Pf(A10)  Pf(A11)  Pf(A12)  Pf(A13)  Pf(A14)  Pf(A15)  Pf(A16)  Pf(B1)  Pf(B2)  Pf(B3)  Pf(B4)  Pf(B5)  Pf(B6)  Pf(B7)  Pf(B8)  Pf(B9)  Pf(B10)  Pf(B11)  Pf(B12)  Pf(B13)  Pf(C1)  Pf(C2)  Pf(C3)  Pf(C4)  Pf(C5)  Pf(C6)  Pf(D1)  Pf(D2)  Pf(D3)  Pf(D4)  Pf(D5)  Pf(D6)  Pf(D7)  Pf(D8)  Pf(D9)  Pf(E1)  Pf(E2)  Pf(E3)  Pf(E4)  Pf(E5)  Pf(E6)  Pf(E7)  Pf(E8)  Pf(E9)  Pf(E10)  Pf(E11)  Pf(E12)  Pf(E13)  Pf(F1)  Pf(F2)  Pf(F3)  Pf(F4)  Pf(F5)  Pf(G1)  Pf(G2)  Pf(I1)  Pf(J1)  Pf(J2)  Pf(M1)  Pf(M2)  Pf(N1)  Pf(N2)  Pf(N3)  Pf(O1)  Pf(O2) | Angola  Nigeria  Democratic Republic of the Congo  Republic of the Congo  Equatorial Guinea  Ghana  Gabon  Cameroon  Zambia  Sierra Leone  Uganda  Côte d'Ivoire | Nanjing  Nanjing  Nanjing  Xuzhou  Xuzhou  Changzhou  Xuzhou  Changzhou  Changzhou  Changzhou  Suzhou  Suzhou  Suzhou  Suzhou  Nantong  Nantong  Nanjing  Xuzhou  Nanjing  Nanjing  Changzhou  Nanjing  Nanjing  Nanjing  Nanjing  Changzhou  Changzhou  Suzhou  Nanjing  Nanjing  Wuxi  Xuzhou  Nanjing  Wuxi  Nanjing  Wuxi  Lianyungang  Nantong  Nanjing  Xuzhou  Changzhou  Nantong  Suzhou  Nantong  Xuzhou  Changzhou  Nantong  Xuzhou  Xuzhou  Xuzhou  Suzhou  Suzhou  Suzhou  Xuzhou  Nantong  Huaian  Xuzhou  Wuxi  Nantong  Yancheng  Nanjing  Nantong  Nanjing  Suzhou  Xuzhou  Suzhou  Nanjing  Changzhou  Lianyungang  Lianyungang  Nantong  Lianyungang  Nanjing  Nanjing | 60500  30545  27500  150000  52377  15783  19666  1031  108925  92411  1140  154622  134180  22811  25410  36280  4250  20300  600  286  10200  16421  320000  12468  6540  19218  18920  84322  32067  28000  98548  3200  230060  102562  1680  124500  64000  155628  15200  126588  1381  9851  1086  155628  18000  1887  204000  24000  5221  2400  68538  12350  51684  1000000  195200  100540  88562  68923  6500  28947  92541  69347  143378  132443  4000000  89538  164339  100800  49187  3200  114982  156824  95411  89546 | MZ444622  MZ444625  MZ444622  MZ444622  MZ444626  MZ444623  MZ444622  MZ444623  MZ444622  MZ444623  MZ444622  MZ444625  MZ444623  MZ444625  MZ444623  MZ444627  MZ444625  MZ444626  MZ444624  MZ444626  MZ444624  MZ444624  MZ444628  MZ444627  MZ444626  MZ444624  MZ444629  MZ444624  MZ444629  MZ444628  MZ444627  MZ444629  MZ444628  MZ444631  MZ444633  MZ444631  MZ444630  MZ444633  MZ444632  MZ444630  MZ444632  MZ444634  MZ444636  MZ444637  MZ444634  MZ444635  MZ444636  MZ444635  MZ444639  MZ444637  MZ444641  MZ444639  MZ444638  MZ444642  MZ444640  MZ444638  MZ444641  MZ444640  MZ444642  MZ444643  MZ444645  MZ444643  MZ444648  MZ444646  MZ444647  MZ444644  MZ444655  MZ444652  MZ444653  MZ444649  MZ444650  MZ444654  MZ444651  MZ444656 |
