# Supplementary material for: Genetic Diversity Analysis of Surface-Related Antigen (SRA) in Plasmodium falciparum Imported From Africa to China
Source: Front Genet. 2021 Aug 5;12:688606. doi: 10.3389/fgene.2021.688606 (PMC8378275; doi:10.3389/fgene.2021.688606)
Supplement: Supplementary Table 2 — Neutrality tests of pfsra among 74 P. falciparum isolates. [file Table_2.DOCX]

[**Supplementary**](https://www.ncbi.nlm.nih.gov/pmc/articles/PMC7645038/#ST1) **Table 2. Neutrality tests of *pfsra* among 74 *P.falciparum* isolates.**

| Country | n | *dN*±SE | *dS*±SE | *dN*/*dS* | Tajima’s *D* | FU & Li’s *D** | FU & Li’s *F** |
| --- | --- | --- | --- | --- | --- | --- | --- |
| Angola  Nigeria  Democratic Republic of the Congo  Republic of the Congo  Equatorial Guinea  Ghana  Gabon  Cameroon  Zambia  Sierra Leone  Uganda  Côte d'Ivoire  Total | 16  13  6  9  13  5  2  1  2  2  3  2  74 | 0.002±0.004  0.003±0.001  0.005±0.002  0.004±0.002  0.005±0.004  0.002±0.003  0.004±0.002  0.003±0.003  0.004±0.004  0.002±0.002  0.005±0.005  0.002±0.002  0.001±0.001 | 0.001±0.003  0.002±0.002  0.001±0.002  0.003±0.001  0.003±0.002  0.001±0.002  0.002±0.001  0.000±0.000  0.004±0.004  0.001±0.001  0.003±0.003  0.001±0.001  0.001±0.002 | 1.523  1.500  5.000  1.333  1.667  2.000  2.000  /  1.000  2.000  1.667  2.000  1.365 | -2.340  -1.642  -1.171  -1.686  -1.856  -1.132  /  /  /  /  -0.738  /  -2.754 | -3.086  -1.804  -0.958  -1.763  -2.255  -1.226  /  /  /  /  -0.738  /  -6.859 | -3.037  -1.847  -0.974  -1.776  -2.257  -1.211  /  /  /  /  -0.738  /  -6.256 |
