# Supplementary material for: Genetic Diversity Analysis of Surface-Related Antigen (SRA) in Plasmodium falciparum Imported From Africa to China
Source: Front Genet. 2021 Aug 5;12:688606. doi: 10.3389/fgene.2021.688606 (PMC8378275; doi:10.3389/fgene.2021.688606)
Supplement: Supplementary Table 3 — The sra Gene ID number and gene length of other Plasmodium species. [file Table_3.DOCX]

**[Supplementary](https://www.ncbi.nlm.nih.gov/pmc/articles/PMC7645038/#ST1) Table 3. The *sra* Gene ID number and gene length of other *Plasmodium* species.**

| Species | Strain | Gene ID from PlasmoDB | Gene length |
| --- | --- | --- | --- |
| *P.falciparum* | 3D7 | PF3D7_1431400 | 2973bp |
| *P.berghei* | ANKA | PBANKA_1013400 | 2454bp |
| *P.chabaudi* | Chabaudi | PCHAS_1014200 | 2424bp |
| *P.gallinaceum* | 8A | PGAL8A_00303000 | 2466bp |
| *P. knowlesi* | strain H | PKNH_1327300 | 2703bp |
| *P. reichenowi* | CDC | PRCDC_1430700 | 2976bp |
| *P. relictum* | SGS1-like | PRELSG_1319700 | 2463bp |
| *P.vivax* | P01 | PVP01_1318000 | 2853bp |
| *P.yoelii* | Yoelii 17X | PY17X_1014900 | 2616bp |
| *P.malariae* | UG01 | PmUG01_13028800 | 2967bp |
| *P.ovale* | curtisi GH01 | PocGH01_13028800 | 2667bp |
